# SALL4 Oncogenic Function in Cancers: Mechanisms and Therapeutic Relevance

**DOI:** 10.3390/ijms23042053

**Published:** 2022-02-12

**Authors:** Boshu Sun, Liangliang Xu, Wenhui Bi, Wen-Bin Ou

**Affiliations:** Zhejiang Provincial Key Laboratory of Silkworm Bioreactor and Biomedicine, College of Life Sciences and Medicine, Zhejiang Sci-Tech University, Hangzhou 310018, China; 2019339902095@mails.zstu.edu.cn (B.S.); 201920201073@mails.zstu.edu.cn (L.X.); 202020801002@mails.zstu.edu.cn (W.B.)

**Keywords:** SALL4, oncogenic function, mechanisms, malignant cancers

## Abstract

SALL4, a member of the SALL family, is an embryonic stem cell regulator involved in self-renewal and pluripotency. Recently, SALL4 overexpression was found in malignant cancers, including lung cancer, hepatocellular carcinoma, breast cancer, gastric cancer, colorectal cancer, osteosarcoma, acute myeloid leukemia, ovarian cancer, and glioma. This review updates recent advances of our knowledge of the biology of SALL4 with a focus on its mechanisms and regulatory functions in tumors and human hematopoiesis. SALL4 overexpression promotes proliferation, development, invasion, and migration in cancers through activation of the Wnt/β-catenin, PI3K/AKT, and Notch signaling pathways; expression of mitochondrial oxidative phosphorylation genes; and inhibition of the expression of the Bcl-2 family, caspase-related proteins, and death receptors. Additionally, SALL4 regulates tumor progression correlated with the immune microenvironment involved in the TNF family and gene expression through epigenetic mechanisms, consequently affecting hematopoiesis. Therefore, SALL4 plays a critical oncogenic role in gene transcription and tumor growth. However, there are still some scientific hypotheses to be tested regarding whether SALL4 is a therapeutic target, such as different tumor microenvironments and drug resistance. Thus, an in-depth understanding and study of the functions and mechanisms of SALL4 in cancer may help develop novel strategies for cancer therapy.

## 1. Introduction

In the past 20 years, molecular targeted therapy and biological cellular immunotherapy have become popular therapeutic methods for tumors [1]. To improve the efficacy of therapy and the patients’ prognosis, ongoing development of novel targets is necessary to develop new drugs and combination therapeutic strategies.

Spalt-like transcription factors (SALLs) are relevant to the occurrence, progression, and prognosis of various tumors, such as cervical cancer and renal cancer [2,3,4,5,6]. SALL1, SALL2, and SALL3 are generally downregulated, while SALL4 is highly expressed in tumors [2]. Hypermethylation of SALL1 and SALL2 are correlated with reduced disease-free survival (DFS) in patients with early neck squamous cell carcinoma (HNSCC) [4] and in patients with oral cancer [7], respectively. SALL3 plays a potential tumor suppressor role in cervical cancer [8,9], whereas *SALL4* is an oncogene in this cancer [10]. Increasing evidence indicates that SALL4 could be a potential target for tumor treatments and a clinically diagnosed idiosyncratic biomarker in different tumors [2,6]. Functions of SALLs in tumors are noted in Table 1. 

Oncofetal SALL4, a zinc finger transcription factor [10,18], is an embryonic stem cell regulator involved in self-renewal and pluripotency [21]. *SALL4* located in chromosome 20.q13.2 translates into two isoforms, SALL4A and SALL4B [10,22], which are a result of different internal splicing patterns in exon 2 [6,22,23]. SALL4A has four zinc finger clusters (ZFCs), three ZFCs of which contain either a pair or a trio of C_2_H_2_-type zinc fingers [22]. Recent studies have found the patterns of SALL4 to bind DNA-specific genes and regulate their expression, involved in ZFC, both in normal embryonic stem cells (ESCs) and SALL4-dependent tumors. SALL4 can bind to short AT-rich motifs via C_2_H_2_ ZFC4 in ESCs, then preventing differentiation [24]. The C-terminal ZFC of SALL4 is responsible for DNA binding, and SALL4 negatively regulates expression of a family of histone 3 lysine 9-specific demethylases (KDMs) in aggressive liver cancer cells [25]. Normally, except in germ cells, SALL4 is silenced in most adult tissues [25]. However, SALL4 was found to be overexpressed in various tumors, such as gastric cancer (GC) [26], lung cancer [15], endometrial cancer [27], hepatocellular carcinoma (HCC) [28], and acute myeloid leukemia (AML) [29]. Additionally, a recent study found that downregulation of SALL4 triggered pluripotency loss, leading to derepression of a set of AT-rich genes that promoted neuronal differentiation [24]. SALL4 is a potential target for the diagnosis and treatment of cancer [18,30]. It participates in the regulation of cell growth, cell cycle, and apoptosis via the expression of articulation-related genes, and it exerts biological effects as a transcription factor in the nucleus, where it plays a crucial role in the occurrence and development of various malignant tumors. Thus, understanding the functions and mechanisms of SALL4 can provide novel insight into how SALL4 can be targeted in tumor therapies.

## 2. Mechanisms of Action of SALL4 in Tumors

Since there are many abbreviations mentioned in this review, we list all the abbreviations in Table 2.

### 2.1. SALL4 Activates the Wnt/β-Catenin Signaling Pathway

Various studies have shown that SALL4 significantly dysregulates the Wnt/β-catenin signaling pathway in cancers. The expression of SALL4 was found to be positively correlated with that of tribbles pseudokinase 3 (TRIB3) in GC [21], which activated Wnt/β-catenin signaling by interacting with the β-catenin–TCF4 complex [31]. In addition, TRIB3 activated the Wnt-β-catenin signaling pathway via the C-terminal and N-terminal regions of the kinase-like domain in TRIB3, directly binding to β-catenin in colorectal cancer [32], indicating that activation of Wnt/β-catenin signaling by SALL4 may require TRIB3 expression. Moreover, TRIB3 knockdown decreased β-catenin expression and resulted in downregulation of c-Myc, axis inhibition protein (AXIN2) and cyclin D1 [31]. In addition, in cervical cancer, SALL4 recognizes and binds to the *CTNNB1* promoter region as a transcription activator and accelerates the expression of β-catenin to upregulate downstream target genes, including *c-Myc* and *CCND1* [10]. Then, the activation of Wnt/β-catenin signaling promotes cell proliferation and tumor growth in cervical cancer cells [10]. SALL4 also regulates the tumorigenicity of breast cancers by modulating the Wnt3a/β-catenin signaling pathway [16]. Furthermore, upregulation of the mRNA and protein of SALL4, Wnt3a, and β-catenin is associated with tumor differentiation in HCC [19].

**Figure 1:**
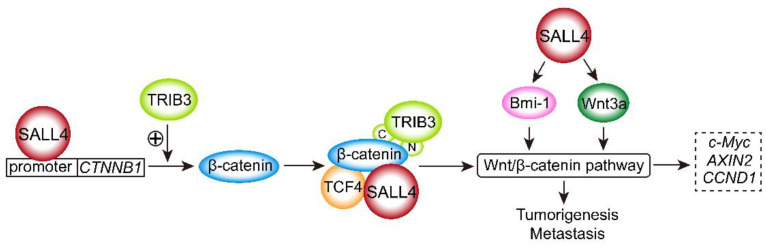
SALL4 activates the Wnt/β-catenin signaling pathway. (1) SALL4 binds to the *CTNNB1* promoter region as a transcription activator and accelerates the expression of β-catenin. (2) The β-catenin–TCF4 complex binds the C-terminal and N-terminal of TRIB3, and then recruits SALL4. (3) SALL4 promotes the expression of Wnt3a and Bmi-1. Through the above-mentioned three ways, SALL4 activates the Wnt/β-catenin signaling pathway and upregulates downstream target genes *c-Myc*, *AXIN2*, and *CCND1*, resulting in tumorigenesis and metastasis.

The SALL4A and SALL4B isoforms have been found to bind to the β-catenin protein in *SALL4B* transgenic mice, and the interactions of these factors activate the Wnt/β-catenin pathway synergistically, which plays essential roles in controlling leukemia stem cell (LSC) self-renewal [33,34,35,36,37]. Downregulation of SALL4 contributed to a decrease in B-cell-specific Moloney murine leukemia virus integration site 1 (Bmi-1) expression and inactivated the Wnt3a/β-catenin signaling pathway in intrahepatic cholangiocarcinoma (ICC) [38]. SALL4 expression was positively correlated with lymph node metastasis and tumor–node–metastasis (TNM) stages in colorectal cancer, and β-catenin was expressed markedly higher in colorectal cancer than in normal tissue [39]. Patients with colorectal cancer with coexpression of SALL4 and β-catenin showed advanced lymph node metastasis and TNM stage. Colocalization of SALL4 and β-catenin was also found in the nucleus and cytoplasm [39]. These findings indicated that the interaction of SALL4 with β-catenin plays crucial functional roles in promoting lymph node metastasis and advanced clinical stage. Another study found that SALL4 modulated the stemness of esophageal squamous cell carcinoma (ESCC) via the Wnt/β-catenin signaling pathway and epithelial–mesenchymal transition (EMT). SALL4 promotes intrahepatic cholangiocarcinoma cell proliferation by activating Wnt/β-catenin signaling [19]. The regulation of Wnt/β-catenin signaling pathway by SALL4 is shown in Figure 1.

### 2.2. SALL4 Inhibits the Expression of PTEN and Activates the PI3K/AKT Signaling Pathway

Phosphatase and tension homolog (PTEN), a tumor suppressor, is expressed at low levels in various cancers [40,41,42]. The well-known PI3K/AKT signaling pathway functions as a key regulator in tumorigenesis [43]. PTEN, as an antagonist of PI3K [44], inhibits AKT activation [45]. High SALL4 expression resulted in proliferative, invasive, and anti-apoptotic effects in lung cancer through activation of PI3K/AKT/mTOR signaling [46]. Other studies found crosstalk between PTEN and SALL4 [47,48,49]. SALL4 silencing resulted in PTEN upregulation, which inhibited PI3K/AKT signaling, indicating that PTEN is a critical functional downstream target of SALL4 in tumor development [50].

**Figure 2:**
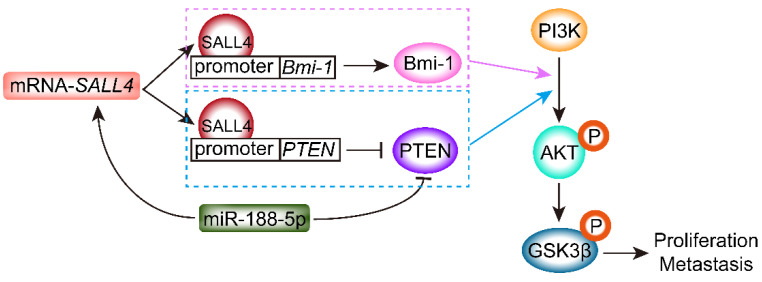
SALL4 regulates tumor proliferation and metastasis through inhibition of PTEN expression and activation of the PI3K/AKT signaling pathway. SALL4 can simultaneously bind to the promoter regions of *Bmi-1* and *PTEN*, leading to increased Bmi-1 expression and decreased PTEN expression, and then activates PI3K/AKT/GSK3β signal pathway. Additionally, miR-188-5p inhibits the expression of PTEN and increases SALL4 transcription.

Furthermore, SALL4 functions as an oncogene associated with other factors, such as Bmi-1 [51], miR-188-5p [52], and deacetylase [53]. As shown in Figure 2, overexpression of Bmi-1 was found to be positively correlated with SALL4 in AML [54]. Downregulation of SALL4 inhibited Bmi-1 expression, while overexpression of SALL4 induced Bmi-1 expression in transgenic mice [51]. SALL4 negatively regulates PTEN expression and positively regulates Bmi-1 expression by binding their promoter, and then activates the PI3K/AKT signaling pathway in hematological tumors [38]. An additional report showed that SALL4 knockdown increased PTEN expression, inhibited downstream intermediate phosphorylation of AKT and GSK3β, and decreased vascular endothelial growth factor A (VEGFA) expression in clear cell renal cell carcinoma (ccRCC), providing convincing evidence that SALL4 is functionally critical in tumor progression and may be regarded as a drug target [55]. A recent study found that miR-188-5p promoted GC cell proliferation and migration by suppressing PTEN expression and transcriptional upregulation of SALL4 [52]. Additionally, positive SALL4 and high histone deacetylase (HDAC) 1/2 expression were correlated with low PTEN expression and a poor prognosis in HCC patients [53].

### 2.3. SALL4 Activates the Notch Signaling Pathway

The Notch signaling pathway plays a critical role in stem cell self-renewal and cell differentiation, and Notch is also involved in the progression and self-renewal capacity of various tumors [56,57]. Ectopic expression of Notch3 increased the expression of the embryonic stem cell marker SALL4 [58].

**Figure 3:**
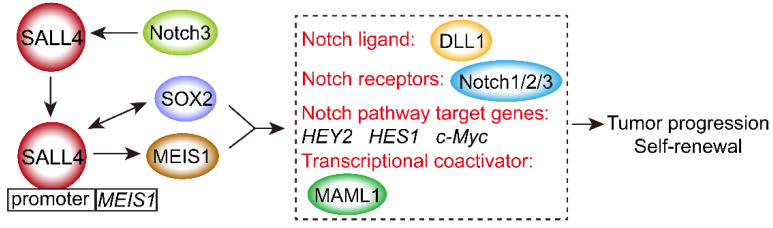
SALL4 activates the Notch signaling pathway. Co-overexpression of SOX2 and SALL4 results in tumor progression and cancer cells self-renewal via activation of the Notch signaling pathway through elevating the expression of main four types of signaling molecules, including Notch ligand DLL1; receptors Notch1/2/3; Notch pathway target genes *HEY2*, *HES1*, and *c-Myc*; and transcriptional coactivator MAML1. Upregulation of Notch3 also increases the expression of SALL4. Moreover, SALL4 induces MEIS1 expression by binding to its promoter, then alters the function of Notch signaling pathway.

Sex-determining region Y (SRY)-Box 2 (SOX2), a partner of SALL4, is a transcription factor regulating cell fate decisions and stem cell self-renewal and pluripotency in ESCs [59]. Concomitant overexpression of SALL4 and SOX2 is relevant to invasion and metastasis in ESCC [60]. However, the mechanisms by which SOX2 and SALL4 promote self-renewal associated with the Notch pathway are still unclear. As shown in Figure 3, current evidence shows that co-overexpression of SOX2 and SALL4 results in upregulation of Notch ligand such as delta-like 1 (DLL1); receptors Notch1/2/3, which induce expression of helix-loop-helix transcriptional repressor target genes hairy/enhancer of split related to YRPW motif family member 2 (*HEY2*); *HES1* and *c-Myc*; and transcriptional coactivators such as mastermind-like transcriptional coactivator 1 (MAML1) in ESCCs. The expression patterns of the Notch pathway genes are similar to those of the mRNA levels of SOX2 and SALL4 [57]. These studies suggest correlations between SOX2/SALL4 expression and the Notch signaling pathway.

Intriguingly, transgenic activation of a mutated *β-catenin* allele was found in murine osteoblasts [61,62] and it induced myelodysplastic syndrome (MDS) and AML at very early ages via dysregulated Notch ligand JAG1 [63,64,65]. Since *SALL4B* transgenic mice also develop MDS/AML and SALL4B-overexpressing bone marrow (BM) cells do not induce leukemia formation in transplanted mice [29], an interesting question is whether/how SALL4B potentially activates β-catenin signaling, which synergistically dysregulates the hematopoietic stem/progenitor cell (HSC/HPC) osteoblastic niche and thus promotes leukemogenesis. Additional detailed transgenic studies are required to address this possibility.

Furthermore, myeloid ecotropic viral insertion site 1 (MEIS1) has been reported as a homeobox transcription factor involved in cell fate decisions and stem cell properties [66,67]. Expression of MEIS1 was associated with SALL4 in poorly differentiated cancer cells. SALL4 induced MEIS1 expression by binding to the promoter region of *MEIS1* [68]. MEIS1 dysregulation may alter the function of the Notch pathway [69,70]. MEIS1 has a close correlation with the stemness state and the Notch signaling pathway involved in SALL4 [71].

Overall, SALL4 can interact with other transcription factors to regulate the Notch pathway or directly interact with Notch and then impact the progression and self-renewal capacity of various cancer cells.

### 2.4. SALL4 Regulates Expression of Bcl-2 and Bax

Apoptosis is regulated by the balance of B-cell lymphoma 2 (Bcl-2) and Bcl-like-protein 4 (Bax) [72]. Overexpression of antiapoptotic Bcl-2 inhibits cell apoptosis and leads to an imbalance between enhanced proliferation and weakened programmed death through downregulation of Bax [73,74]. SALL4 overexpression in MDS and AML cells results in an increase in Bcl-2 expression and cell survival [75], whereas SALL4 knockdown markedly inhibits Bcl-2 expression in prostate cancer [76], breast cancer [16,77], colorectal cancer (CRC) [78], and nasopharyngeal carcinoma (NPC) [79], and it induces the expression of pro-apoptotic Bax in prostate cancer [76] and NPC [79].

### 2.5. SALL4 Inhibits Caspase-Related and Death-Receptor Pathways

SALL4 not only regulates cell growth, such as the Wnt/β-catenin and PTEN/PI3K/AKT pathways [21,50], but also affects cell apoptosis via the caspase-related and death-receptor pathways. A study showed that SALL4 regulated the expression of apoptosis genes and apoptotic process-related genes such as *CYC3* and *CUL3* [80]. SALL4 knockdown in AML cells resulted in upregulation of apoptotic genes, including *CARD9*, *CARD11*, *TNF*, *ATF3*, *TP53*, *PTEN*, and *LTA*, but downregulation of antiapoptotic genes, such as *Bmi-1*, *Bcl-2*, *DAD1*, *BIRC4*, *BIRC7*, and *TEGT* [80]. Furthermore, a previous study showed that Bmi-1 was one of the major downstream targets of SALL4 in leukemia [51]. SALL4 restored Bmi-1 knockdown-induced cell cycle arrest and apoptosis [80].

**Figure 4:**
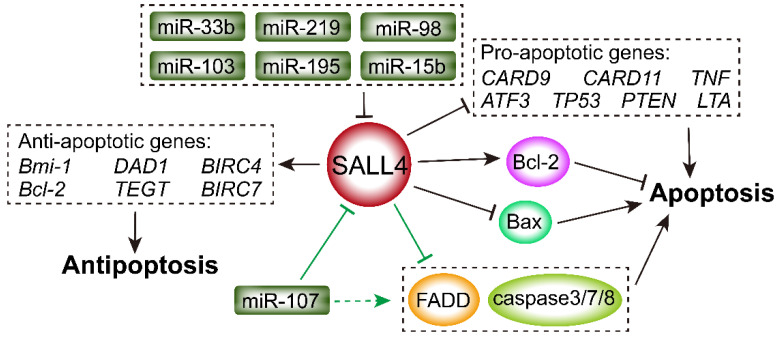
SALL4 regulates Bcl-2, Bax, caspase-related pathway, and death-receptor pathway. MiRNAs (miR-33b, miR-219, miR-98, miR-103, miR-195, miR-15b, and miR-107) suppress SALL4 expression. MiR-107 induces cell apoptosis by directly targeting SALL4, resulting in the increased expression of FADD and the activation of caspase-8 and caspases-3/7. SALL4 contributes to cell survival by suppression of apoptotic genes such as *Bax*, *CARD9/11*, *TNF*, *ATF3*, *TP53*, *PTEN*, and *LTA*, as well as activation of anti-apoptotic genes including *Bmi-1*, *DAD1*, *BIRC4*, *Bcl-2*, *TEGT*, and *BIRC7*.

SALL4 influences tumor survival by regulating multiple caspase members. SALL4 knockdown induced the expression of caspase-3 and caspase-8 in adult acute B cell lymphoblastic leukemia (B-ALL) [81]. Moreover, various microRNAs (miRNAs) play tumor suppressor roles, such as miRNA-98 and miRNA-33b in HCC and miRNA-219 in glioma by inhibiting SALL4 expression and suppressing the proliferation and metastasis of tumor cells [82,83,84,85]. miR-103, miR-195, and miR-15b have identical 5′ miRNA sequences and share common binding sites in the 3′-untranslated region (UTR) of *SALL4*, which inhibits SALL4 expression and activates caspase-3 and caspase-7 in glioma cells [86]. Furthermore, miRNA-107 suppressed glioma cell growth by directly targeting SALL4, resulting in the increased expression of Fas-associated by death domain (FADD) and the activation of caspase-8 and caspases-3/7, inducing cell apoptosis [14]. The correlations between SALL4 and Bcl-2, Bax, caspase-related pathway, and death-receptor pathway are shown in Figure 4.

### 2.6. MiRNAs Regulate the Interaction of SALL4 and TNF Family

**Figure 5:**
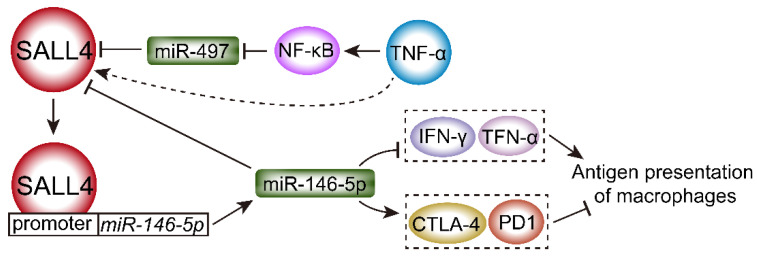
MiRNAs regulate the interaction of SALL4 and TNF family. (1) TNF-α/NF-κB induces SALL4 expression by suppression of miR-497, promoting self-renewal and metastasis phenotypes. (2) SALL4 reduces the antigen presentation ability of macrophages through directly binding the promoter and inducing expression in miR-146a-5p, which inhibits the expression of IFN-γ and TNF-α and increases the expression of PD-1 and CTLA-4.

Cancer progression is closely related to the modulation of the tumor microenvironment [87]. Tumor necrosis factor alpha (TNF-α), a proinflammatory cytokine, has been reported to be important in the development and progression of HCC by activating the transcription factor nuclear factor κB (NF-κB) [88]. In the tumor microenvironment, it is possible that the antitumor immune response can be switched to a protumor response [89]. During tumor progression, T-cell-mediated antitumor response is significantly impaired, characterized by low secretion of interleukin-2 (IL-2), TNF-α, and interferon-γ (IFN-γ) and a high expression of inhibitory receptors such as PD-1 [90]. As shown in Figure 5, miR-497 negatively regulates SALL4 expression and then inhibits metastasis and self-renewal in HCC. TNF-α, as an inflammatory factor, suppresses miR-497 expression by NF-κB-mediated negative transcriptional regulation and upregulates SALL4 expression, promoting self-renewal and metastasis phenotypes in HCC [91]. Furthermore, SALL4 could bind to the promoter of miR-146a-5p and directly regulate its expression in exosomes. HCC-derived exosomes could remodel macrophages by activating NF-κB signaling and inducing proinflammatory factors.

Additionally, SALL4 promotes the expression of miR-146a-5p by binding its promoter, while silencing SALL4 inhibits the expression of inhibitory receptors and reverses T-cell exhaustion (Figure 5). HCC cell-secreted miR-146a-5p can be delivered by exosomes into macrophages, switch the cytokine profile, and attenuate antigen presentation of macrophages through inhibiting the expression of IFN-γ and TNF-α and upregulating the expression of inhibitory receptors such as programmed death ligand 1 (PD-1) and cytotoxic-T-lymphocyte-antigen-4 (CTLA-4), promoting the production of alternatively activated M2-polarized tumor-associated macrophages [87] (Figure 5).

### 2.7. SALL4 Regulates Gene Expression through Epigenetic Mechanisms

**Figure 6:**
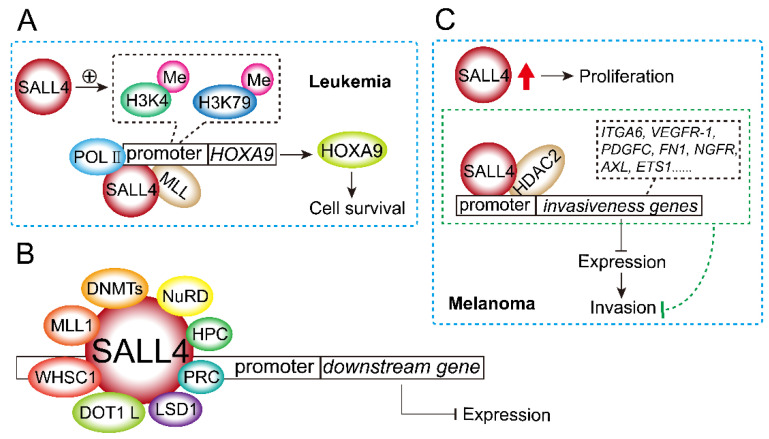
SALL4 regulates gene expression through epigenetic mechanisms. (**A**) SALL4/MLL/HOXA9 pathway is a crucial regulator of leukemic cell survival. SALL4 interacts with POLII and MLL, co-occupies the HOXA9 promoter region with MLL, and then increases HOXA9 expression. Moreover, overexpression of SALL4 enhances methylation of histone H3K4 and H3K79. (**B**) Epigenetic factors that interact with SALL4 include NuRD complex, DNMTs, MLL1, WHSC1, DOT1 L, lysine-specific histone demethylase LSD1, HPC, and PRC to bind the promoter of downstream gene and regulate their expression. (**C**) Although SALL4 overexpression results in melanoma proliferation, SALL4 inhibits invasiveness genes by interacting with HDAC2 and directly binding to invasive genes including *NGFR*, *ETS1*, *VEGFR-1*, and *PDGFC*. Red arrow indicates an increase of SALL4 expression.

SALL4 regulates gene expression through epigenetic mechanisms. As shown in Figure 6A, SALL4 has been found to interact with histone methyltransferase mixed lineage leukemia (MLL) and co-occupy the homeobox A9 (*HOXA9*) promoter region with *MLL*. Moreover, overexpression of SALL4 enhances markers of histone activation, such as H3K4 and H3K79 methylation, as well as RNA polymerase II (POLII) binding in the same promoter region, which increases HOXA9 expression in AML. The data demonstrated that the SALL4/MLL/HOXA9 pathway was a crucial regulator of leukemic cell survival [92]. In addition, SALL4 interacts directly with DNA methyltransferases (DNMTs), indicating that SALL4 can inhibit transcription by recruiting DNA methyltransferases [93]. SALL4 also interacted with histone lysine-specific demethylase 1 (LSD1) to inhibit gene transcription in stem cells [94]. Furthermore, SALL4 co-occupies target genes with the polycomb repressive complex (PRC), indicating that SALL4 may inhibit gene transcription by inducing PRC components such as Bmi-1 or by interacting with PRC members, including RNF2, PhcI, EZH2, EED, SUZ12, and RbAp46/48, as well as HPC proteins [6,95].

Regulatory malignancies of SALL4 are involved in multiple chromatin modification regulators [29]. As previously reported, epigenetic factors that interact with SALL4 include six types: the nucleosome remodeling deacetylase (NuRD) complex, which includes HDAC1 and HDAC2 [49,96]; DNA methyltransferases-1 (DNMT-1), DNMT-3A, DNMT-3B, DNMT-3 L, and methyl-CpG-binding domain 2 protein (MBD2) [18]; H3K4 methyltransferase MLL1 [92,97]; H3K36 methyltransferase Wolf–Hirschhorn syndrome candidate gene-1 (WHSC1) [98,99]; H3K79 methyltransferase disruptor of telomeric silencing 1-like (DOT1 L) [68,100]; and lysine-specific histone demethylase LSD1/KDM1A [49,94,101] (Figure 6B). Interestingly, SALL4 seems to interact with these epigenetic factors at different sites. For instance, while the amino-terminal 174 amino acid sequence of SALL4 is critical for the SALL4–DNMT1 or SALL4–HDAC interaction, it is less relevant to the SALL4–LSD1 interaction. This is important in designing drugs to block protein–protein interaction-based anti-SALL4 strategies. Notably, clinical epigenetic remedies inhibiting SALL4-interacting epigenetic factors such as DOT1 L, HDAC, and LSD1 have been shown to be effective in treating leukemia [102,103,104,105,106] Figure 6B). Cutaneous melanoma is the most aggressive skin cancer owing to its high invasiveness [107], and melanoma cells rely on developmental programs during tumor initiation and progression [18]. Diener et al. found that SALL4 negatively regulated invasiveness by interacting with HDAC2 and directly binding to invasiveness genes, including *NGFR*, *ETS1*, *FN1*, *VEGFR-1*, and *PDGFC* (Figure 6C). SALL4 knockdown with HDAC inhibition promoted an invasive phenotype, while inhibition of histone acetylation partially reversed the invasiveness program induced by SALL4 depletion [18]. Thus, SALL4 was identified as a regulator of melanoma phenotype switching from tumor formation (SALL4 overexpression) to invasiveness (SALL4 loss). These epigenetic factors are not only significant regulators of normal hematopoiesis but are also targets for dysregulation in malignancies, especially in hematological tumors such as leukemia [108,109,110,111].

SALL4 is one of the significant factors required for the enhancement and maintenance of HSC/HPCs [29]. Overexpression of SALL4 in mobilized peripheral blood CD34^+^ cells promoted ex vivo expansion efficiency by over 10,000-fold for CD34^+^/CD38^−^ and CD34^+^/CD38^+^ cells with appropriate cytokines [112]. Similarly, the SALL4B isoform expanded BM-derived CD34^+^ nonhuman primate HSCs. SALL4 lentiviral transduction caused a sixfold change in the total cell count of CD133^+^ HSCs compared to the control group [113,114]. Additionally, Milanovich et al. thought that SALL4 might be important for murine hematopoiesis [115].

SALL4 interacts with acetylase to induce phenotypic conversion. SALL4 is well known as an ESC regulator [116], and SALL4 knockdown induces a phenotype switch and an invasive phenotype via an HDAC2-mediated mechanism [18] (Figure 6C). SALL4 expression is highly correlated with DNA promoter methylation. SALL4, HDAC1, and DNMT proteins co-occupy the same promoter sites of SALL4, and their own transcription may be downregulated via recruitment of both DNA methylation and histone deacetylation enzymes [93,117]. The mRNA expression of DNA methyltransferase 3 alpha (DNMT3A) in the SALL4 knockdown group was markedly downregulated, indicating that DNMT3A may contribute to the regulatory effects of SALL4 [117].

Overall, the function of SALL4 involves many epigenetic mechanisms, and it inhibits or activates gene transcription through epigenetics. SALL4 not only plays a critical role in normal hematopoiesis through such an epigenetic mechanism but is also a common target in leukemia and many malignant tumors.

### 2.8. SALL4 Induces Mitochondrial Oxidative Phosphorylation during Tumorigenesis

**Figure 7:**
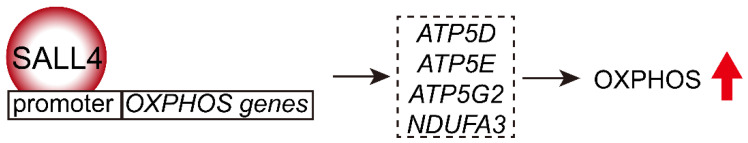
SALL4 induces mitochondrial OXPHOS during tumorigenesis in HCC. SALL4 drives tumorigenesis by binding to the promoter of OXPHOS genes and transcriptionally activating the expression of these genes, and then increases mitochondrial OXPHOS (Red arrow indicates an increase of OXPHOS).

A recent study found that SALL4 induced mitochondrial oxidative phosphorylation (OXPHOS) during tumorigenesis [118] (Figure 7). A phenomenon commonly called the Warburg effect usually occurs in during tumorigenesis [119]. Cancer cells usually rewire their bioenergetic metabolism by changing from mitochondrial OXPHOS to aerobic glycolysis, despite the presence of oxygen. SALL4-positive cells resist this common trend in HCC cell metabolism by driving mitochondrial OXPHOS instead. SALL4 induction of OXPHOS and mitochondrial ATP leads to the allosteric suppression of basal glycolysis rates [118]. Thus, SALL4-positive cancer cells possess a latent capacity for the Warburg effect, but this latent capacity is suppressed in the steady state. Additionally, SALL4 overexpression increases the transcription of mitochondrial OXPHOS genes, and SALL4 silencing leads to downregulation of OXPHOS genes, such as *ATP5D*, *ATP5E*, *ATP5G2*, and *NDUFA3*, as well as a functional reduction in mitochondrial oxygen consumption and OXPHOS [118] (Figure 7).

Although there are several transcription factors that act as oncogenes, few drugs have been developed to inhibit their activity in tumors [120,121,122]. However, a recent study showed that SALL4 increased OXPHOS, mitochondrial membrane potential, oxygen consumption rate, and utilization of oxidative phosphorylation-related metabolites to generate ATP [123]. The ATP synthase inhibitor oligomycin reduced the viability of HCC and lung cancer cells with high SALL4 expression. These results indicate that inhibitors of OXPHOS may be used for the treatment of HCC with high levels of SALL4.

Overall, SALL4 drives tumorigenesis by binding to and transcriptionally activating OXPHOS genes or expressing high levels of SALL4 by blocking OXPHOS to reduce tumor growth, providing novel therapies for HCC. However, an extension of the understanding of cancer metabolic reprogramming is necessary to push the development of innovative therapies.

### 2.9. Brief Summary of Mechanisms of SALL4 in Cancers

Specific epigenetic signatures, gene expression patterns, and signaling pathways contribute to the establishment and maintenance of stemness state networks. For one thing, SALL4 is an underlying biomarker that activates signaling pathways and oncoproteins that promote tumor progression including Wnt/β-catenin (Figure 1), PI3K/AKT (Figure 2), Notch signaling pathway (Figure 3), and OXPHOS (Figure 7), as well as Bcl-2, TNF-α, and IFN-γ (Figure 5). Moreover, SALL4 regulates gene expression through epigenetic mechanisms, then affecting tumor progression (Figure 6). For example, SALL4/MLL/HOXA9 pathway is a crucial regulator of leukemic cell survival and SALL4 plays a critical role in promoting melanoma invasion by interacting with HDAC2. For another, SALL4 can also inhibit apoptotic proteins, miRNAs, or pathways to promote tumor development, such as miRNAs, PTEN, Bax, caspase-related pathway, and so on (Figure 4). Overall, as an oncogene, SALL4 plays a pivotal role in carcinogenesis in different types of normal and tumor tissues.

## 3. Function of SALL4 in Tumors

### 3.1. Lung Cancer

Lung cancer is one of the most malignant tumors causing cancer-related deaths, with its mortality ranking high among malignancies in China and worldwide; lung cancer is classified into non-small cell lung cancer (NSCLC) and small cell lung cancer (SCLC), and the former represents approximately 85% of all lung cancer cases [124,125,126]. The therapies for lung cancer are chemotherapy, resection, and radiotherapy, but the prognosis is still unsatisfactory, with an overall 5-year survival rate of only 15% for all stages [124,127,128]. Additionally, immunotherapy and molecular targeted therapy have been applied in lung cancer clinical treatment to increase the survival rates [15,129]. A previous study showed that SALL4 expression is abnormally high in lung cancer [130], indicating that a deep understanding of the function underlying the progression and prognosis of lung cancer involved in SALL4 is critical for promoting an early diagnosis and effective therapy. Downregulation of SALL4 inhibits cell proliferation, clonal formation, migration, and invasion of lung cancer while promoting cell arrest in the G0/G1 phase by inhibiting the expression of cell cycle-related proteins such as cyclin B, cyclin D1, and cyclin E [131].

SALL4 regulates the progression of lung cancer through several mechanisms, and it may have a close correlation with drug resistance. SALL4 was upregulated in lung cancer with *EGFR* mutations [132]. Activation of EGFR could induce SALL4 overexpression by the extracellular signal-regulated kinase 1/2 (ERK1/2) signaling pathway, while SALL4 knockdown could not only inhibit spheroid formation and the expression of CD44, a type of lung cancer stem cell marker, but also increase the sensitivity of EGFR-mutated cells to EGFR inhibitor erlotinib [15]. Furthermore, knockdown of SALL4 can restore cisplatin sensitivity in acquired resistant lung cancer cells via the AKT/mTOR signaling pathway [46], and the antineoplastic drug entinostat can target SALL4-positive lung cancer [133]. SALL4 can also interact with miRNAs in lung cancer, including miR-250 and miR-3619 [134]. Upregulation of SALL4 and homeobox A11 antisense (HOXA11-AS) inhibited miR-3619-5p expression, whereas HOXA11-AS knockdown led to pro-apoptotic and anti-proliferative, -migration, -invasion, and glycolysis effects. Moreover, miR-3619-5p attenuates the inhibitory effects of HOXA11-AS knockdown on the progression of NSCLC cells by directly binding to HOXA11-AS. SALL4 overexpression reversed the antitumor effect of miR-3619-5p by decreasing miR-3619-5p expression. HOXA11-AS silencing inhibited tumor growth via upregulation of miR-3619-5p and downregulation of SALL4 [135].

### 3.2. Hepatocellular Carcinoma

Liver cancer, the sixth most common cancer, is the second leading cause of cancer mortality worldwide due to limited therapeutic interventions [136]. Liver cancer includes hepatoblastoma (HB) and HCC; the former is a common type of liver cancer reported in infants under 3 years of age, and the latter is the most common type of liver cancer documented, affecting 85–90% of liver cancer patients [118,137,138,139]. SALL4 is reactivated in HCC, where 30–50% of tumors show high SALL4 expression [140]. Since HCC patients can only receive palliative treatments, including chemotherapy and pain relief [141], there are still some challenges for improving HCC therapy. Thus, a better understanding of the function of SALL4 may help patients acquire a novel therapeutic strategy.

SALL4 regulates HCC progression by interacting with miRNAs. miR-15a inhibited cell proliferation, migration, and invasion in HCC by downregulating SALL4 [142]. Furthermore, miR-497 directly targeted SALL4 to inhibit SALL4 expression and the self-renewal and metastasis of HCC [91]. Meanwhile, another study showed that TNF-α inhibited miR-497 expression through NF-κB-mediated negative transcriptional regulation as well as upregulation of SALL4, promoting the self-renewal and metastasis phenotypes of HCC cells [91]. SALL4 mediates miR-146-5p expression in exosomes by binding its promoter. Suppression of the SALL4 and miR-146a-5p interaction decreases the expression of inhibitory receptors on T cells and reverses apoptosis, delaying HCC progression [87].

Additionally, SALL4 can be regarded as a therapeutic target for HCC patients. SALL4 can not only activate the Wnt/β-catenin signaling pathway but also mediate PTEN silencing together with HDAC1/2 and then promote HCC development, leading to a poor prognosis [19,53]. SALL4 can activate the transcription of genes that regulate OXPHOS to increase oxygen consumption, mitochondrial membrane potential, and ATP generation in HCC. Thus, targeting OXPHOS might be used for the treatment of liver tumors with high SALL4 expression [118]. Demethylation of CpGs located within octamer-binding transcription factor 4 (OCT4) and activator of transcription 3 (STAT3) cis-acting elements downstream of the *SALL4* transcriptional start site TSS (*SALL4*TSS) enables OCT4 and STAT3 binding, recruitment of BRG1, and enhanced RNA polymerase II elongation and *SALL4* transcription, inducing *SALL4* re-expression in HCC associated with hepatitis B or C virus infection [143].

In addition, SALL4 can also be a diagnostic marker for liver cancer. Evaluation of SALL4 and LIN28, a type of RNA-binding protein, increased the accuracy of distinguishing classic gastric hepatoid carcinomas (GHCs) from HCCs [144]. SALL4 serves as a diagnostic marker of subtyping of HB for the cytology and the prognostication of patients [145].

### 3.3. Breast Cancer

Breast cancer (BC) represents a leading cause of cancer-related death in the female population around the world, causing 14% of all cancer-related deaths [146,147,148]. Current therapeutic methods, including immunotherapy, molecular targeted therapy, cell therapy, radiotherapy, chemotherapy, and surgery, increase patient survival rates [149]. Nevertheless, challenges such as immune escape, drug resistance, and poor apoptosis urgently need to be resolved [150].

With increasing attention given to targeting SALL4 in the treatment of BC, it has been found that SALL4 knockdown results in cell cycle arrest and reversal of chemoresistance in BC cells by downregulating BC resistance proteins [151]. SALL4 knockdown reduced cell proliferation, migration, and invasion in BC cells [152] and induced cell cycle arrest in G0/G1 phase and apoptosis, which might be mediated by downregulating the Wnt/β-catenin pathway [16]. Moreover, SALL4 promotes tumor migration and mammosphere formation in vitro and tumorigenicity in vivo in BC by inducing mesenchymal markers such as vimentin by directly binding to its promoter [153]. Some studies have shown that SALL4 is an important survival marker in static lymph nodes relative to the primary site in BC [153,154]. Tripartite motif-containing 21 (TRIM21), identified as an E3 ubiquitin-protein ligase, is involved in nuclear SALL4 degradation. TRIM21 localizes in the nucleus in BC cells, and TRIM21 knockdown increases the SALL4B-EGFP probe by TRIM21 targeting Lys-190, suggesting that SALL4 is polyubiquitinated at Lys-190 and degraded in BC [155].

Basal-like BC is an aggressive cancer with no effective treatment thus far [156]. CD44, a membrane protein, is a stemness factor in cancer, and its splicing variants are involved in cancer stemness [157,158]. SALL4 modulates CD44 alternative splicing by regulating a splicing factor for CD44 (KHDRBS3), enhancing anoikis resistance in basal-like BC [159]. Furthermore, *SALL4* was highly expressed in tamoxifen-resistant (TAMR) patients and had a positive correlation with *Oct4*, *Nanog*, and *SOX2* stemness markers, suggesting that SALL4 overexpression contributed to TAMR and poor survival rates in tamoxifen-treated BC patients [154]. Thus, targeting SALL4 could be a novel strategy for clinical treatment of BC.

Overall, SALL4 is involved in the positive regulation of BC cell progression. The development of therapies targeting SALL4 may address the problem of drug resistance and provide new therapeutic strategies for aggressive BC.

### 3.4. Gastric Cancer

Gastric cancer (GC) ranks third in tumor-related mortality worldwide [160]. Although recent therapeutic advancements have decreased the mortality and incidence of GC, the prognosis is still poor, and the 5-year survival rate is lower than 30% [161,162,163]. Overexpressed SALL4 has been found to be an indicator of a poor prognosis in GC [164,165]. SALL4 regulates GC progression by several mechanisms. (1) SALL4 upregulation activates oncogenic Wnt signaling to promote GC tumorigenesis by interacting with the transcriptional mechanisms of *TRIB3* [21]. (2) SALL4 exerts its oncogenic activities in GC cells by activating DANCR and the β-catenin pathway [166]. DANCR is a lncRNA that is required for the suppression of epidermal cell differentiation, which is associated with advanced tumor progression and a poor prognosis [167,168]. (3) SALL4 overexpression leads to EMT and metastasis in GC by activating the TGF-β/SMAD signaling pathway [20]. (4) SALL4 promotes GC progression via hexokinase II (HK-2)-mediated glycolysis [26]. However, HK-2 knockdown reversed the promoting role of SALL4 in GC cell proliferation, migration, and invasion [20,166,169], indicating that SALL4 drives GC progression by upregulating HK-2 [26].

MiR-188-5p promoted GC proliferation and migration, and inhibited tumor suppressor PTEN expression by transcriptional upregulation of SALL4. SALL4 knockdown eliminated the proliferative effect of miR-188-5p and increased PTEN expression [52]. Additionally, overexpression of miR-16 suppressed cell viability and migration via SALL4 downregulation [170].

### 3.5. Colorectal Cancer

Colorectal cancer (CRC) is one of the most common and lethal cancers, leading to approximately 8% of all tumor-related deaths [127,171]. Not only does CRC have a high mortality rate and a poor prognosis if there is metastasis to the lymph nodes [172], but it also has two challenges, therapeutic resistance and tumor relapse [32]. SALL4 is overexpressed in the majority of CRCs and plays an oncogenic role in CRC progression, maintenance, and metastasis [173]. Thus, understanding the exact underlying mechanisms and exploring new diagnostic and therapeutic markers for CRC is urgently needed. SALL4 silencing markedly reduced the proliferation, invasiveness, and drug resistance of CRC via inhibition of Gli1 [174]. Moreover, SALL4 and β-catenin colocalized and interacted with each other in promoting lymph node metastasis and an advanced clinical stage [39]. Additionally, *SALL4* mRNA was highly increased in the blood of CRC patients, indicating that SALL4 can be a potential biomarker for early detection of CRC [175]. However, there are still few studies on treating CRC by targeting SALL4.

### 3.6. Osteosarcoma

Osteosarcoma (OS) is one of the most common primary high-grade bone malignancies, frequently occurring in the metaphysis of long bones, mainly in adolescents and young adults [125]. The estimated incidence of this tumor is 5 per million annually in China [176]. Unfortunately, OS patients still suffer from a low survival rate because of metastatic lesions and drug resistance [177,178,179]. Thus, SALL4 as a therapeutic candidate target in OS has been considered. SALL4 knockdown not only inhibited OS cell proliferation, invasion, and migration in vitro and suppressed OS growth and metastasis in vivo but also inhibited Wnt/β-catenin signaling [180]. Additionally, SALL4 expression was upregulated by sponging miR-107, contributing to OS growth and invasion [17]. These studies show that SALL4 regulates the progression of OS cells and may facilitate the identification of new predictive indicators and targets for OS treatment. However, there are still few studies on SALL4 as a target in the treatment of OS, and more in-depth studies on the molecular mechanism and clinical applications are needed.

### 3.7. Normal Hematopoietic Function and Leukemia

Leukemia is a type of malignancy in heterogeneous HSCs that is characterized by aberrant accumulation of undifferentiated blasts capable of unrestrained proliferation in the bone marrow and it interferes with the production of normal blood cells [181]. Leukemia is mainly divided into four subtypes, namely, chronic myeloid leukemia (CML), chronic lymphoblastic leukemia (CLL), acute lymphoblastic leukemia (ALL), and AML, the latter being one of the most lethal cancers [182].

AML has a poor prognosis [183] and is a clonal disorder arising from the differentiation arrest of myeloid precursors and the malignant proliferation of bone marrow-derived, self-renewing stem or progenitor cells in the BM and blood [184]. Clinical sequelae generally include discomfort and fatigue, infection, bleeding and/or bruises, and often bone pain [185]. Acute promyelocytic leukemia (APL) is a type of AML [186], and a heterocyclic compound, indole, is considered an attractive candidate for cancer therapy [187]. A previous study has found that indole inhibits the expression of *SALL4* mRNA in APL cells and that SALL4 can be regarded as a target in APL [187]. SALL4 is positively related to Bmi-1, which is expressed at markedly higher levels in AML patients [188]. *SALL4* mRNA is highly expressed in AML, while it is little expressed in normal hematopoiesis [33].

Additionally, *SALL4* expression was higher among AML patients with CD34-positive expression than among those with CD34-negative expression, which may have a close correlation with a high capacity for self-renewal [183]. SALL4 has different effects on both proapoptotic and antiapoptotic pathways in normal and leukemic cells [189]. In the process of normal hematopoiesis, *SALL4* is preferentially expressed in human CD34-positive hematopoietic stem/progenitors and downregulated in CD34-negative cells during hematopoietic differentiation [33]. Aberrantly, SALL4 was expressed in primary leukemia, AML, and precursor B-cell lymphoblastic leukemia [184]. Furthermore, SALL4 expression correlates with disease progression in human CML, and its expression in AML patients correlates with treatment status [190]. SALL4 expression patterns in AML and CML patients during different disease progression phases and its relationship to patient survival and risk stratification have been explored.

### 3.8. Ovarian-Related Diseases

Ovarian cancer (OC) is the fifth cause of cancer-related death among women, and around 21,410 new OC patients and the mortality rate of 13,770 female patients of OC have been reported in 2021 according to the American Cancer Society [191]. Epithelial ovarian carcinoma (EOC) is the most common OC, a serous ovarian carcinoma (SOC), accounting for 95% of ovarian malignancies, which is diagnosed at advanced stages of disease in about 70% cases due to non-specific sign or symptoms of ovarian tumors [192,193]. In clinical diagnosis, there are some common biomarkers for screening high-risk OC females, including carcinoembryonic antigen (CEA), ova1, human epididymis protein 4 (HE4), overa, and risk of ovarian malignancy algorithm (ROMA) [194]. However, these biomarkers have low accuracy and sensitivity [195]. Thus, optimal biomarkers with high sensitivity and accuracy for OC prognosis are needed.

The mRNA expression of *SALL4* and aldehyde dehydrogenase 1 (*ALDH1*) has been found to different in SOC tissues. Specifically, *SALL4* was upregulated while *ALDH1A1* was downregulated [196]. Clinical study in OC patients showed the same patterns of protein expression of SALL4 and ALDH1A1 [197,198]. The analysis of expression of the two markers has been determined, but the combination or interaction between these two markers has remained elusive. Moreover, co-expression of SALL4 and ALDH1A1 is associated with more aggressive tumor behavior, advanced disease, and poor disease-specific survival (DSS), or progression-free survival (PFS) in SOC cases, and higher co-expression of SALL4/ALDH1A1 was found as an independent prognostic factor for PFS [196]. The application of SALL4/ALDH1A1 may be the sensitive or accurate biomarkers for OC prognosis, but further investigations with more patients are needed to verify above results.

Recently, *SALL4* was reported as a target of gene therapy in premature ovarian insufficiency (POI) [199]. POI is a severe female disorder characterized by primary or secondary amenorrhea before 40 years of age [200]. *SALL4* is involved in cell growth and development and it is a reasonable target for gene therapy. It is worth mentioning that POI may be seen in patients with a mutated *SALL4* gene [201]. Wang et al. observed that compared to wild-type SALL4, all three SALL4 missense variants (1790A>G, 541G>A, and 2279C>T) identified in POI subjects significantly increased the SALL4 protein expression, leading to enhanced SALL4 regulatory activity [202]. These missense variants maybe increase protein stability, with posttranslational regulation, suggesting the essential genetic involvement of *SALL4* in the etiology of POI. However, caution must be taken, since SALL4 is known to function as an oncogene in various death-related tumors [20,134,142,152].

### 3.9. Glioma

Glioma is the most common highly malignant primary brain tumor, representing more than 80% of cancers in the brain [203,204]. Although there are some improvements in the currently available therapeutic interventions, the prognosis of patients is still poor. Identification of the new targets that lead to glioma is critical for anticancer drug development [205].

Previously, miRNAs have been reported as oncogenes or tumor suppressors in the glioma cells [14]. MiR-30a-5p has been found to promote glioma cell growth by targeting septin 7 [206], but miR-124 inhibits the migration and invasion of glioma cells by directly suppressing the expression of Rho-associated protein kinase 1 [207]. After that, SALL4 was gradually identified as a novel target of miRNAs. For example, SALL4 was negatively regulated by miR-219 and miR-181b in glioma cells, suggesting that miR-219 and miR-181b act as a suppressive role in glioma growth and metastasis via targeting SALL4 [85,208]. Although the function of SALL4 in glioma was determined, the regulatory mechanism of miRNA/SALL4 in glioma remained elusive. Aiming at investigating the specific mechanism, Chen et al. explored the correlation between miRNAs and SALL4, finding that miR-103, miR-195, and miR-15b all had the same 5′ “seed” miRNA portion and shared common binding sites in the SALL4 3′-UTR. In addition, the caspase-3/7 expression in glioma cells overexpressing these miRNAs was rescued during SALL4 upregulation [86], indicating that miR-103, miR-195, and miR-15b inhibit glioma cell growth, migration, and invasion through post-transcriptional downregulation of SALL4. Moreover, high methylation decreased miR-98 expression, further promoting migration and invasion in glioma via targeting SALL4 [209]. SALL4 knockdown also induced cell cycle arrest, enhanced early apoptosis, and inhibited invasion in glioma, which was associated with a markedly low expression of the core transcription factors, including POU class 5 homeobox 1; SOX2; and Nanog homeobox in glioblastoma multiforme (GBM), a subtype of glioma [210]. Additionally, SALL4 acts as an oncoprotein by suppression of PTEN expression and activation of PI3K/AKT signaling pathway, thereby facilitating proliferation of glioma cells [50]. Overall, SALL4 serves a crucial role in the glioma pathophysiology and may be a potential approach to the treatment of glioma by regulating miRNAs expression and PTEN/PT3K/AKT signaling pathway.

## 4. Analysis and Prospects in the Future

This review summarizes the mechanisms and function of SALL4 in lung cancer [131], ovarian cancer [197], cervical cancer [211], renal cancer [2], colon adenocarcinoma [175], breast cancer [155], osteosarcoma [180], and so on, indicating that SALL4 is a promising and potential biological marker for cancer diagnosis and a therapeutic target. SALL4 participates in tumor growth, invasion, and migration in diverse cancers and is involved in the Wnt/β-catenin, Notch, PTEN/PI3K/AKT pathways, TNF family, and some caspase-related proteins. SALL4 can be used not only as a diagnostic biomarker or therapeutic target in cancer but also as a drug resistance marker. Furthermore, SALL4 plays a critical role in both normal hematopoiesis and many malignant tumors through epigenetic mechanisms and activation of OXPHOS genes, providing novel therapies for cancer.

However, there were some limitations of our data source, and additional studies are required to establish best practices for minimizing unwanted batch effects while preserving biological signals. For example, SALL4 expression is related to p53 and the IAP family, but the specific mechanism has not been determined. The pathological role of SALL4 in cancer seems to depend on the cell type and microenvironment. Therefore, it is necessary to establish a mouse model in which the SALL4 subtype is conditionally activated or knocked out in some cell types [6]. Nevertheless, additional studies to exploit the potential of SALL4 for cancer therapy are still valuable and promising.

## Figures and Tables

**Table 1:** Function of different types of SALL family members in tumors.

SALL4 Family Member	Function in Tumors	Mechanism	References
SALL1	Anti-proliferative effects in glioma	Inhibit Wnt/β-catenin signaling	[11]
	Induce angiogenesis	Active the *VEGF-A* gene	[12]
SALL2	Tumor suppressor	Inhibit *c-Myc* gene	[13]
SALL3	Promote tumorigenesis	*SALL3* promoter hypermethylation	[3]
SALL4	Oncogene in glioma	Induce epithelial–mesenchymal transition	[14]
	Promote progression in lung cancer	Activate EGFR by ERK1/2 signaling	[15]
	Promote proliferation, migration, and invasion in breast cancer	Activate Wnt/β-catenin signaling	[16]
	Promote growth and invasion in osteosarcoma	Sponge miR-107	[17]
	Promote invasiveness in melanoma	Interact with the HDAC 2 and bind to invasiveness genes	[18]
	Promote development in hepatocellular carcinoma	Activate Wnt/β-catenin signaling	[19]
	Promote metastasis in gastric cancer	Activate the TGF-β/SMAD signaling	[20]

**Table 2:** Abbreviations.

Abbreviation	Full Name
ALDH1	aldehyde dehydrogenase 1
ALL	acute lymphoblastic leukemia
AML	acute myeloid leukemia
APL	acute promyelocytic leukemia
AXIN2	axis inhibition protein
B-ALL	B-cell lymphoblastic leukemia
Bax	Bcl-like-protein 4
BC	breast cancer
Bcl-2	B-cell lymphoma 2
BM	bone marrow
Bmi-1	B cell-specific Moloney murine leukemia virus integration site 1
ccRCC	clear cell renal cell carcinoma
CEA	carcinoembryonic antigen
CLL	chronic lymphoblastic leukemia
CML	chronic myeloid leukemia
CRC	colorectal cancer
CTLA-4	cytotoxic-T-lymphocyte-antigen-4
DFS	disease-free survival
DLL1	delta-like 1
DNMTs	DNA methyltransferases
DNMT3A	DNA methyltransferase 3 alpha
DOT1 L	disruptor of telomeric silencing 1-like
DSS	disease-specific survival
EMT	epithelial–mesenchymal transition
EOC	epithelial ovarian carcinoma
ERK1/2	extracellular signal-regulated kinase 1/2
ESCC	esophageal squamous cell carcinoma
ESCs	embryonic stem cells
GBM	glioblastoma multiforme
GC	gastric cancer
GHCs	gastric hepatoid carcinomas
HB	hepatoblastoma
HCC	hepatocellular carcinoma
HDAC	high histone deacetylase
HE4	human epididymis protein 4
HEY2	hairy/enhancer of split related to YRPW motif family member 2
HK-2	exokinase II
HNSCC	neck squamous cell carcinoma
*HOXA9*	homeobox A9
HOXA11-AS	homeobox A11 antisense
HPC	hematopoietic progenitor cell
HSC	hematopoietic stem cell
ICC	intrahepatic cholangiocarcinoma
IFN-γ	TNF-α and interferon-γ
IL-2	interleukin-2
KDMs	histone 3 lysine 9-specific demethylases
LSD1	lysine-specific demethylase 1
MAML1	mastermind-like transcriptional coactivator 1
MBD2	methyl-CpG-binding domain 2 protein
MDS	myelodysplastic syndrome
MEIS1	myeloid ecotropic viral insertion site 1
miRNA/miR	microRNA
MLL	mixed lineage leukemia
NF-κB	transcription factor nuclear factor κB
NPC	nasopharyngeal carcinoma
NSCLC	non-small cell lung cancer
NuRD	nucleosome remodeling deacetylase
OC	ovarian cancer
OCT4	octamer-binding transcription factor 4
OS	osteosarcoma
OXPHOS	oxidative phosphorylation
PD-1	programmed death ligand 1
PFS	progression-free survival
POI	premature ovarian insufficiency
POLII	RNA polymerase II
PRC	polycomb repressive complex
PTEN	phosphatase and tension homolog
ROMA	risk of ovarian malignancy algorithm
SALLs	spalt-like transcription factors
*SALL4*TSS	*SALL4* transcriptional start site TSS
SCLC	small cell lung cancer
SOC	serous ovarian carcinoma
SOX2	sex-determining region Y (SRY)-Box 2
STAT3	activator of transcription 3
TAMR	tamoxifen-resistant
TNF-α	tumor necrosis factor alpha
TNM	tumor node metastasis
TRIB3	tribbles pseudokinase 3
TRIM21	tripartite motif-containing 21
UTR	untranslated region
WHSC1	Wolf–Hirschhorn syndrome candidate gene-1

## Data Availability

Not applicable.
